# Early identification of the nosocomial spread of vancomycin-resistant *Enterococcus faecium* by Fourier-transform infrared spectroscopy and performance comparison with PFGE and WGS

**DOI:** 10.1080/22221751.2024.2392659

**Published:** 2024-08-13

**Authors:** Cristina Pitart, Maria Piquet, Tessa Burgwinkel, Rocío Arazo Del Pino, Marc Rubio, Mireia Aguilar, Sergi De Gea, Andrea Pulgarín, Irene Campo, Blanca Torralbo, Romina Parejo, Silvia Valls, Isabel Fortes, Gemina Santana, Elisa Rubio, Anna Vilella, Ana Del Río, José Antonio Martínez, Elisenda Miró, Ferran Navarro, Mateu Espasa, Climent Casals-Pascual, Jordi Vila, Paul G. Higgins, Ignasi Roca

**Affiliations:** a Department of Microbiology, Biomedical Diagnostic Center (CDB) and ISGlobal, Hospital Clínic – Universitat de Barcelona, Barcelona, Spain; b Institute for Medical Microbiology, Immunology and Hygiene, Faculty of Medicine and University Hospital Cologne, University of Cologne, Cologne, Germany; c German Center for Infection Research, Partner site Bonn–Cologne, Cologne, Germany; d Department of Microbiology, Institut d’Investigació Biomèdica Sant Pau, Hospital de la Santa Creu i Sant Pau, Barcelona, Spain; e Department of Genetics and Microbiology, Universitat Autònoma de Barcelona, Barcelona, Spain; f Department of Preventive Medicine and Epidemiology, Hospital Clínic–Universitat de Barcelona, Barcelona, Spain; g Molecular Core Facility, Hospital Clínic of Barcelona, Barcelona, Spain; h Department of Infectious Diseases, Hospital Clínic–IDIBAPS, Universitat de Barcelona, Barcelona, Spain; i CIBER de Enfermedades Infecciosas (CIBERINFEC), Instituto de Salud Carlos III, Madrid, Spain

**Keywords:** Vancomycin-resistance, epidemiology, outbreak, Fourier-transform infrared spectroscopy, strain typing

## Abstract

Early detection of disseminating vancomycin-resistant *Enterococcus faecium* (VREfm) in ICU wards is crucial for outbreak identification and the implementation of prompt infection control measures. Genotypic methods like pulsed-field gel electrophoresis (PFGE) and whole-genome sequencing (WGS) are costly and time-consuming, hindering rapid response due to batch dependency. Fourier-transform infrared spectroscopy (FT-IR) offers the potential for real-time outbreak detection and reliable strain typing. We utilized FT-IR to identify clonal VREfm dissemination and compared its performance to PFGE and WGS. Between February through October 2023, an unusually high number of VREfm were recovered at a tertiary hospital in Barcelona. Isolates were examined for antimicrobial susceptibility, carriage of *vanA/vanB* genes and clonality was also studied using FT-IR, PFGE, and WGS. Routine FT-IR inspections revealed recurring VREfm clustering during the outbreak's initial weeks. In total, 104 isolates were recovered from 75 patients and from multiple wards. However, only one isolate was recovered from an environmental sample, suggesting the absence of environmental reservoirs. An ST80 vancomycin-resistant (*vanA*) *E. faecium* strain was the main strain responsible for the outbreak, although a few additional VREfm strains were also identified, all belonging to CC17. PFGE and cgMLST (WGS) yielded identical clustering results to FT-IR, and WGS confirmed *vanA/vanB* gene carriage in all VREfm isolates. Infection control measures led to a rapid decline in VREfm isolates, with no isolates detected in November. FT-IR spectroscopy offers rapid turnaround times, sensitivity, and reproducibility, comparable to standard typing methods. It proved as an effective tool for monitoring VREfm dissemination and early outbreak detection.

## Introduction

Since they emerged in the USA and Europe in the 1980s, vancomycin-resistant enterococci (VRE) infections have increased worldwide. Vancomycin-resistant *Enterococcus faecium* (VREfm) account for most VRE cases and frequently cause severe infections in immunocompromised patients [1,2]. Hospital-related VREfm isolates tend to be highly clonal, typically belonging to clonal lineages related to clonal complex 17 (CC17) that became vancomycin-resistant mainly due to acquiring the *vanA* or *vanB* glycopeptide resistance determinants [3]. VREfm resistance rates across European countries varies enormously, ranging from 67% in Lithuania, 49% in Greece, 18% in Germany, to a mere 0.3% in Sweden. In Spain the prevalence of VREfm is also very low, with resistance rates around 2.8% usually from sporadic hospital outbreaks [4, 5].

VREfm have become a significant health issue due to their ability to persist and disseminate within the hospital environment, combined with a very narrow antimicrobial susceptibility profile, making it challenging to eradicate them from hospital settings. Not surprisingly, VREfm was included in the high-priority group of pathogens for research and development of new antibiotics issued by the WHO in 2017 [6]. Early detection of disseminating VREfm in ICU wards is therefore crucial for outbreak identification and early response. However, quick determination of clonal spread remains challenging despite the available technologies. DNA analysis by pulsed-field gel electrophoresis (PFGE) has been the gold standard for decades. It provides excellent resolution but lacks interlaboratory reproducibility, has a relatively low sample throughput, it is time-consuming and mostly batch-dependent [7]. Whole genome sequencing (WGS), on the other hand, is the new gold standard due to its high discriminatory and reproducible nature, but it is still expensive and time-consuming, with significant bottlenecks associated with data processing and analysis [7, 8]. Long-read sequencing technologies, like Oxford Nanopore, provide faster turnaround times but are still costly and have accuracy issues, though this is likely to improve in the foreseeable future.

More recently, Fourier-transform infrared (FT-IR) spectroscopy has emerged as a promising spectrum-based technique with great potential applications in clinical microbiology [9]. This technique quantifies the absorption of infrared light related to the vibrational bond energies of molecules, generating an FTIR spectrum that reflects the chemical composition of the sample, thus providing specific fingerprints. FT-IR spectroscopy can be used to compare spectra and classify them according to their molecular similarity, allowing for bacterial strain typing. This has been successfully tested with bacterial isolates of *Escherichia coli, Klebsiella pneumoniae* or *Acinetobacter baumannii*, among others [10]. Here we report the microbiological characterization of an outbreak caused by VREfm in a tertiary hospital in Barcelona and the use of FT-IR in its early detection, evaluating its performance compared to PFGE and WGS.

## Materials and methods

In total the study included 104 *E. faecium* isolates collected from 75 different patients and the hospital environment from February through October 2023 at a tertiary hospital (819 beds) in Barcelona, Spain. A prospective study including up to 10 VREfm isolates was performed to rapidly identify clonal dissemination by FT-IR. A retrospective study was then used to compare the performance of FT-IR, PFGE and WGS.

### Case definition

Any patient admitted to the hospital with an isolate of VREfm recovered either in a clinical or a surveillance sample after 48 h from admission was considered as a case of VREfm infection or colonization, respectively.

### Surveillance and microbiology

While VREfm isolates were extremely rare at our hospital before 2023, with only five and two isolates being reported in 2021 and 2022, respectively, MDR Gram-negative and MRSA were frequently reported. The hospital had a nosocomial infection control program that included active surveillance to detect MDR Gram-negative bacilli and MRSA. It involved nasal and fecal swabbing of all immunosuppressed, ICU, and referred patients upon admission and weekly thereafter, as well as the weekly sampling of all patients in wards with at least one positive patient (either in a clinical or surveillance sample) until no positives were recorded for two consecutive weeks. Additionally, environmental samples using moistened sterile swabs were collected in rooms with at least one patient testing positive during any active outbreak situation. Until January 2023, fecal and environmental surveillance samples were plated only on CHROMID^®^ ESBL and CHROMID^®^ CARBA SMART selective and chromogenic media (Biomérieux, France). CHROMID^®^ VRE plates were also routinely included once the first VREfm isolates were detected.

Colonies growing on CHROMID^®^ plates were identified by matrix-assisted laser desorption ionization-time time of flight mass spectrometry (MALDI-TOF MS) with a Microflex LT benchtop instrument (Bruker Daltonics) operated in linear positive mode. The presence of *vanA*/*vanB* genes was initially assessed using the GeneXpert system (Cepheid, US) and later confirmed by PCR and WGS [11].

Antibiotic susceptibility was assessed using the BD Phoenix™ system (Becton-Dickinson, USA) for ampicillin, vancomycin, teicoplanin, linezolid, imipenem and daptomycin. Interpretation of MIC values followed EUCAST guidelines and breakpoints for *Enterococcus* (European Committee on Antimicrobial Susceptibility Testing, 2023 v.13). *Enterococcus faecalis* ATCC 29212 was used for quality control.

For clonality and WGS studies, *E. faecium* was grown on Tryptic Soy agar (TSA, Condalab, Spain) plates at 37°C in a 5% CO_2_ atmosphere for 24 h.

### PFGE

PFGE was performed from freshly grown bacterial cultures. A 1.5 McFarland solution was used to prepare agarose plugs (1% Seakem Gold agarose, Lonza, Switzerland) for further digestion in lysis buffer (50 mM Tris-HCl, pH = 8; 50 mM EDTA; 1% N-lauroil-sarcosine; 100 μg/ml Proteinase K) and restriction of chromosomal DNA at 25°C with SmaI (New England BioLabs Inc., United States). Fragment separation was achieved on a CHEFF-DR III system (Bio-Rad, Spain) running at 6 V/cm^2^ with switch times ranging from 3.5s to 23.5s at an angle of 120°, for 20 h at 14°C [12]. Molecular patterns were analyzed with InfoQuestTM FP-v.5.4 (Bio-Rad, Spain) and the unweighted pair group method with arithmetic mean (UPGMA) to create dendrograms based on Dice’s similarity coefficient [13]. Bandwidth tolerance and optimization values were set at 1.5% and isolates were considered within the same PFGE cluster (pulsotype) if their Dice similarity index was >85%. Overall, the turnaround time for PFGE including plug preparation, lysis and DNA digestion, electrophoresis and analysis was no less than 3 days.

### FT-IR

For FT-IR spectroscopy, a 1 μl loopful of bacterial colony was dissolved in 70% ethanol and fully homogenized by extensive vortexing with the aid of added small sterile metal cylinders in the Eppendorf tubes. The homogenized samples were further dissolved in 1 volume of sterile water and spotted (15 μl) onto a 96-well FTIR silicon plate and allowed to dry. Five technical replicates and two biological replicates were used for every bacterial sample.

Infrared absorption spectra were captured using a Bruker IR Biotyper instrument (Bruker Daltonic) running on the IR Biotyper software version 4.0 and on default settings (32 scans per technical replicate; spectral resolution, 6 cm^−1^; apodization function, Blackman-Harris 3-term; zero-filling factor, 4). Measurements were carefully checked and spectra that did not meet the quality criteria (0.4 < absorption < 2; signal-to-noise ratio, > 40; fringes [x10^−6^], < 100) were removed. Spectra processing for initial clustering of the isolates was based on the second derivative of the 1300–1800 cm^−1^ region associated with absorption data from cell wall carbohydrates. Captured spectra from technical and biological replicates that met the quality criteria were used to calculate the underlying average spectra for each sample and added to the reference database for hierarchical analyses based on Euclidean average distances and the UPGMA linkage algorithm. Overall, the turnaround time for FT-IR spectroscopy including sample preparation, acquisition and analysis was less than 3 h.

### MLST and cgMLST

Multi-locus sequence typing (MLST) and clonal complexes were determined following the MLST scheme described by Homan *et al.* using genomic sequences as input data at the pubMLST database [14, 15]. Genomic sequences were also investigated using a validated cgMLST scheme (including 1,423 target alleles from the core genome and 1,124 alleles from the accessory genome for a total of 2,547 alleles), using the Ridom SeqSphere + v.9.0.10 software (Ridom GmbH, Germany) to illustrate clonal relatedness [16]. Previous studies have suggested a cut-off of ≤20 alleles to identify *E. faecium* isolates belonging to the same clone [17]. In our experience, however, some STs tend to be highly clonal and a lower threshold is needed in order to differentiate among clonal lineages. Hence, isolates differing in ≤9 alleles were considered closely related and designated a cluster of isolates.

### Whole genome sequencing

Genomic DNA of selected isolates was extracted using the DNeasy UltraClean Microbial kit (Qiagen, Hilden, Germany) following the manufacturer’s instructions. Sequencing libraries were prepared using the Ultra II FS DNA Library Prep Kit (New England Biolabs, Frankfurt, Germany) for a 250-bp paired-end sequencing run on an Illumina MiSeq sequencer as previously described [18]. Reads were assembled de novo using SKESA (version 2.4.0) which is integrated in the Ridom SeqSphere + v.9.0.10 software (Ridom GmbH, Münster, Germany). The raw sequencing reads generated in this project were submitted to the European Nucleotide Archive under the study accession number PRJEB73881.

### Resistome analysis

Presence of antimicrobial resistance determinants as well as chromosomal mutations that confer resistance were investigated using the assembled genomes and querying NCBI AMRFinder (last accessed 1st May, 2024) and the ResFinder (v.4.5.0) website (http://genepi.food.dtu.dk/resfinder).

### Calculation of clustering concordance

The concordance of FT-IR clustering in comparison to PFGE, MLST, and cgMLST typing methods was assessed by calculating the adjusted Rand index (ARI) and adjusted Wallace coefficient (AWC) with 95% confidence interval using the online Comparing Partitions tool (www.comparingpartitions.info).

ARI determines the overall pairwise congruence of different typing methods [19], and AWC compares the agreement of two typing methods considering one of them as the reference method [20]. ARI and AWC values may range from 0 to 1, where 0 means agreement expected by chance and 1 means perfect correlation between both methods [20, 21].

## Results

### Early detection of nosocomial dissemination

On February 3, 2023, a single VREfm isolate was recovered from the rectal swab of a patient admitted to the hepatology ICU. Subsequently, 8, 32, and 29 additional VREfm isolates were recovered in March, April and May, respectively, either from surveillance or clinical samples.

**Figure 1.**
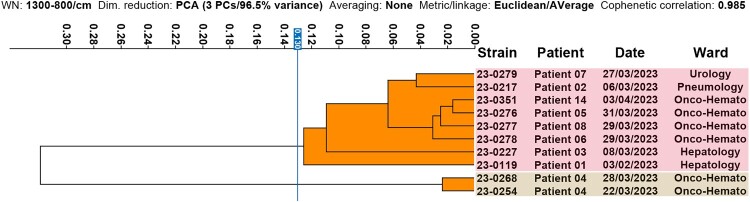
Early outbreak detection. Clustering analysis from the FT-IR spectra of the initial 10 VREfm isolates of the outbreak. The two different clusters are highlighted either in red or in light brown. Isolates were included within the same FT-IR cluster if their Euclidean distance values were ≤1.30 (Blue line).

**Figure 2.**
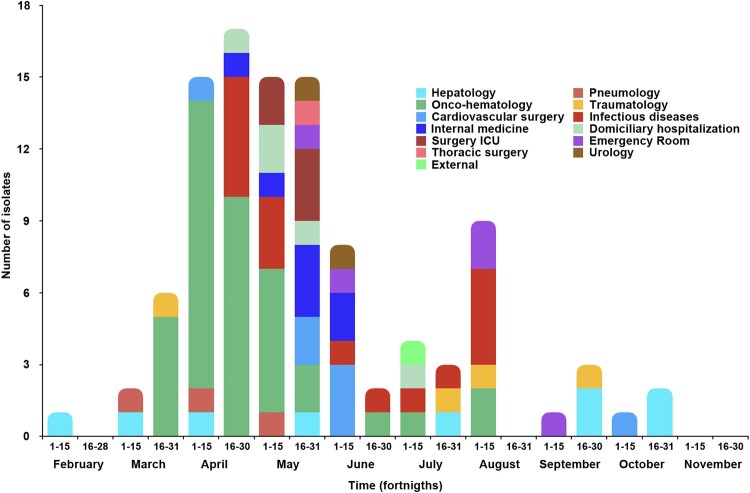
Spatial and temporal distribution of 104 *E. faecium* isolates recovered between February through November 2023 according to ward of isolation.

Early FT-IR analysis of the first 10 VREfm isolates of 2023 at our hospital showed their grouping into just two clusters using a Euclidean distance cut-off value of 0.130. One cluster contained 8 clonally-related isolates recovered from an equal number of patients while the other cluster included just two isolates recovered from the same patient (Figure 1). In view of putative nosocomial dissemination, the swift implementation of infection control measures was recommended. These measures included reinforcing hand hygiene, environmental and equipment cleaning, environmental screening, cohort isolation, and chlorhexidine bathing of patients. Between February and October 2023, as many as 10,412 surveillance swabs (including 481 swabs for environmental screening) were performed throughout the hospital. Dissemination of VREfm was carefully monitored and gradually contained, with only 11 isolates recovered in June, seven in July, nine in August, four in September, and only three isolates in October. No new isolates were recovered from additional patients after October 2023, marking the resolution of the outbreak (Figure 2).

In total, the outbreak involved 75 patients, yielding 104 VREfm isolates, with 96 of them obtained from surveillance samples (including one environmental sample) and eight isolates recovered from clinical samples (wound n = 5; blood n = 2; synovial fluid n = 1) of seven different patients.

Although the first VREfm isolate was recovered from a patient in the hepatology unit, only seven additional patients in this ward were affected throughout the outbreak. Instead, nosocomial dissemination primarily occurred among onco-hematological patients in the first three months of the outbreak, affecting 27 patients (three of them with positive clinical samples). There was also broad dissemination among patients admitted to the infectious diseases ward, involving 13 additional patients with intestinal colonization. Other affected wards included cardiovascular surgery, internal medicine ward, the emergency room, pneumology, and urology wards, as well as a few patients under domiciliary hospitalization but with previous hospital admissions. Unfortunately, the source of the outbreak was never identified.

### Outbreak strain

The microbiological characterization of the isolates showed that the outbreak was caused by the hospital dissemination of a VREfm clonal lineage (designated VRE_1) assigned to ST80 belonging to clonal complex 17 (CC17). VRE_1 isolates carried the *vanA* gene and exhibited resistance to both vancomycin and teicoplanin with MICs >8 mg/L. They were also resistant to ampicillin (MIC >16 mg/L), imipenem (MIC >8 mg/L) and linezolid (MIC >4 mg/L), showing daptomycin MICs ≤2 mg/L. WGS of representative isolates (see below) showed that resistance to linezolid among VREfm isolates in this study was associated with the carriage of the *poxtA* gene encoding an ABC-F type ribosomal protection protein [23]. Mutations in either the 23S rRNA gene or other ribosomal proteins were not detected.

### Clonality studies and performance comparison with PFGE and WGS

A retrospective epidemiological study that included 71 *E. faecium* isolates (54 patients) recovered from February 1st to May 31st was performed to evaluate the performance of FT-IR compared to that of PFGE and WGS (Table S1). Representative isolates from each clonal cluster were also further selected to undergo WGS.

**Figure 3.**
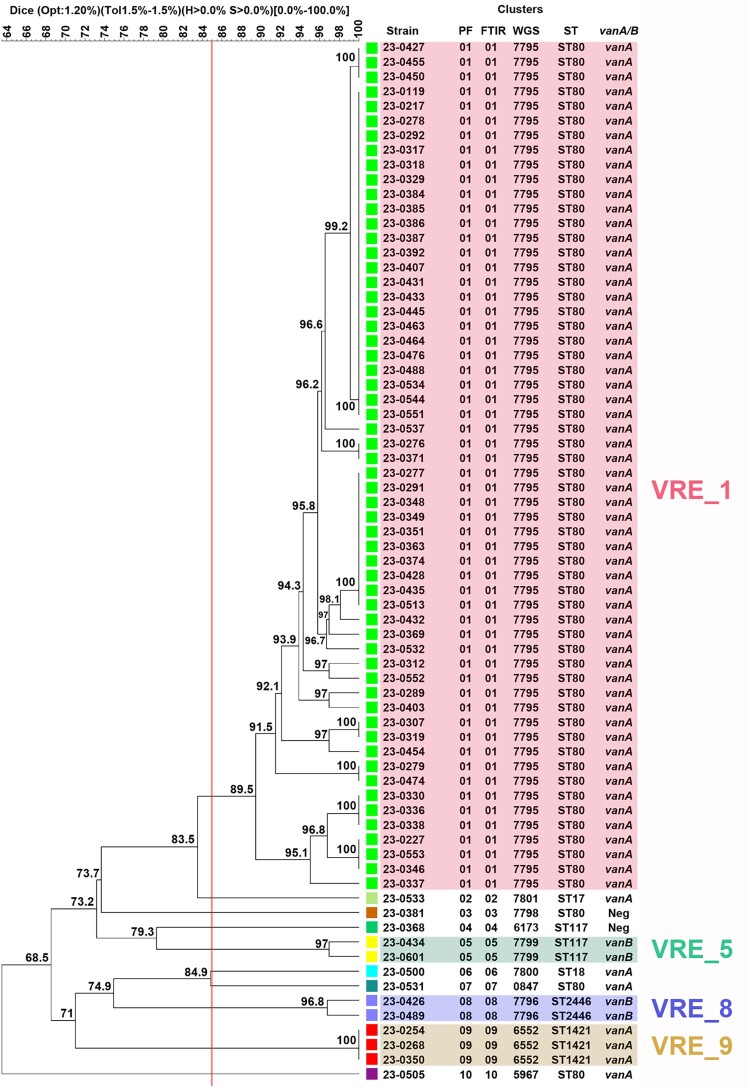
PFGE dendrogram of 71 *E. faecium* isolates recovered from February through May 2023 from a single tertiary hospital in Barcelona. PF: Pulsed-field cluster number; FTIR: FT-IR spectroscopy cluster number; WGS: cgMLST cluster number; ST: Sequence type number; *vanA/B*: Positive for the presence of either the *vanA* or the *vanB* genes. Isolates were included within the same pulsed-field cluster if their Dice similarity index was ≥85% (Red line). Clusters containing more than one isolate are highlighted using different colors: VRE_1 in red, VRE_5 in olive green, VRE_8 in violet and VRE_9 in light brown.

Among the 71 isolates, PFGE analysis identified 10 different clusters (Figure 3). A major cluster (VRE_1) included 58 isolates corresponding to the VanA-producing outbreak strain associated with ST80. This cluster mainly disseminated within the onco-hematology and infectious diseases wards, as mentioned previously. Additional clusters VRE_2, VRE_3, VRE_4, VRE_6, VRE_7, and VRE_10 contained a single isolate each. Strains from clusters VRE_2 and VRE_6 were identified as ST17 and ST18, respectively, while those from clusters VRE_7 and VRE_10 also belonged to ST80. All isolates from these clusters carried the *vanA* gene. Strains from clusters VRE_3 and VRE_4 were associated with ST80 and ST117, respectively, but were not VREfm as they proved susceptible to both vancomycin and teicoplanin and were also negative for *vanA/vanB* screening. Despite not being considered VREfm, these two isolates were kept in the study for comparison purposes.

Cluster VRE_5 included two *vanB*-positive ST117 isolates recovered from two different patients admitted to the onco-hematology ward, suggesting nosocomial transmission. Cluster VRE_8 included two ST2446/*vanB* isolates and cluster VRE_9 included three ST1421/*vanA* isolates, but in both clusters, all isolates originated from the same patient, ruling out nosocomial transmission of these strains. All isolates were assigned to STs belonging to CC17, as previously reported for VREfm hospital isolates [23]. Table 1 shows the microbiological features of a representative isolate from each cluster, as identified by PFGE.

**Table 1.** Microbiological features of the first isolate from each of the 10 different E. faecium clusters identified from February through May 2023 at a single tertiary hospital in Barcelona.

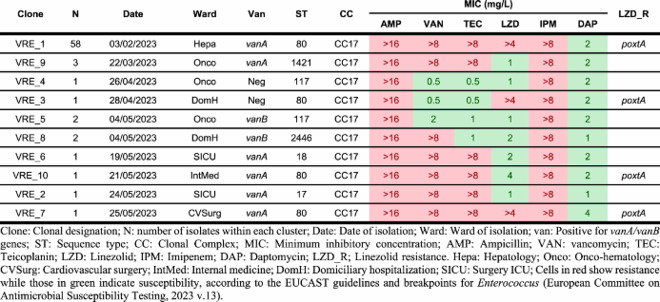

**Figure 4.**
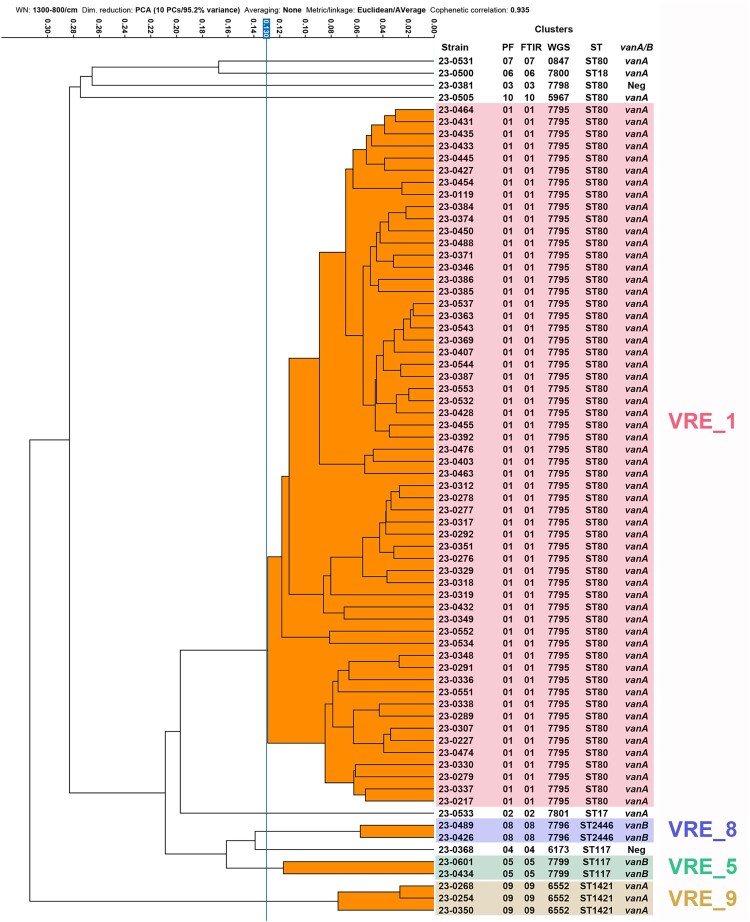
FT-IR dendrogram of 71 *E. faecium* isolates recovered from February through May 2023 from a single tertiary hospital in Barcelona. PF: Pulsed-field cluster number; FTIR: FT-IR spectroscopy cluster number; WGS: cgMLST cluster number; ST: Sequence type number; *vanA/B*: Positive for the presence of either the *vanA* or the *vanB* genes. Isolates were included within the same FT-IR cluster if their euclidean distance values were ≤1.30 (Blue line). Clusters containing more than one isolate are highlighted using different colors: VRE_1 in red, VRE_5 in olive green, VRE_8 in violet and VRE_9 in light brown.

**Figure 5.**
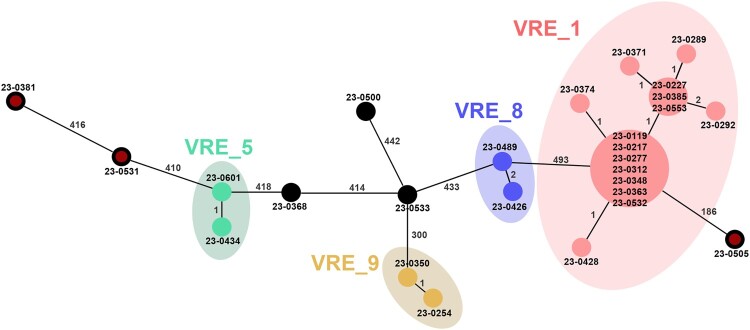
Minimum spanning tree of representative *E. faecium* isolates based on a cgMLST scheme that included 2,547 target alleles (core and accessory genome). The strain designation of the isolates is shown at the nodes and the numbers between nodes indicate the number of alleles that were different. Isolates were included within the same cgMLST cluster if they differed in ≤9 alleles. Nodes representing non-outbreak ST80 strains are shown with a red inner dot. Clusters containing more than one isolate are highlighted using different colors: VRE_1 in red, VRE_5 in olive green, VRE_8 in violet and VRE_9 in light brown.

FT-IR analysis of the same subset of 71 isolates, using a Euclidean distance cut-off of 0.130, also provided 10 different clusters, each containing identical strains as those included within the PFGE clusters (Figure 4). The PFGE clustering results were used to select 27 isolates representing each cluster, including several isolates from the main outbreak clone, that were further studied by WGS. cgMLST analysis based on 2547 targets (core and accessory genome) grouped the selected isolates into 10 different partitions or clusters, once again matching the clustering of all isolates by PFGE and FT-IR. The isolates within the cgMLST clusters, corresponding to the VRE_1, VRE_5, VRE_8, and VRE_9 clusters identified by both FT-IR and PFGE, were either identical or differed by up to three alleles (Figure 5). The congruence of cluster composition yielded ARI and AWC values of 1.000 when considering PFGE as the reference method, thus showing full concordance between all three methods (Tables 2 and 3).

**Table 2.** Adjusted Rand index (ARI) comparing PFGE, FT-IR, MLST and cgMLST.

Adjusted Rand Index	PFGE	FT-IR	MLST	cgMLST
PFGE				
FT-IR	1.000			
MLST	0.828	0.828		
cgMLST	1.000	1.000	0.828

**Table 3.** Adjusted Wallace coefficient (AWC) comparing PFGE, FT-IR, MLST and cgMLST.

Adjusted Wallace Index	PFGE	FT-IR	MLST	cgMLST
PFGE		1.000	1.000	1.000
		(1.000-1.000)	(1.000-1.000)	(1.000-1.000)
FT-IR	1.000		1.000	1.000
	(1.000-1.000)		(1.000-1.000)	(1.000-1.000)
MLST	0.707	0.707		0.707
	(0.397-1.000)	(0.397-1.000)		(0.397-1.000)
cgMLST	1.000	1.000	1.000	
	(1.000-1.000)	(1.000-1.000)	(1.000-1.000)	

On the other hand, MLST analysis only resulted in 6 different partitions, as some partitions included several isolates assigned to the same ST (mainly ST80 and ST117) that nevertheless clustered separately by PFGE (the reference method) as well as by FT-IR and cgMLST. Compared to the other methods, MLST showed a lower degree of cluster congruence with an ARI value of 0.828 (Tables 2 and 3). Similarly, the directional AWC value between MLST and the reference method (MLST→PFGE) was 0.707 with a 95% CI of 0.397-1.000, representing the directional probability of two isolates clustered together by MLST also being clustered by the reference method.

## Discussion

While the percentage of VREfm in Europe has been steadily increasing since 2018, Spain remains one of the 10 European countries with VREfm percentages below 5% [5, 24]. Nevertheless, we report here the rapid dissemination of an ST80 VREfm strain, along with an increase in the number of additional VREfm strains recovered at our hospital compared to previous years.

Overall, at least eight different VREfm strains (including the outbreak strain) from a total of 104 isolates were identified during this period, representing a significant increase compared to only five and two isolates reported in 2021 and 2022, respectively. It is unclear whether this increase is due to a higher prevalence of circulating VREfm strains in our local area or merely reflects an intensification in active surveillance at our hospital triggered by the identification of the outbreak strain. It is important to highlight that VREfm strains in our study were mainly identified colonizing the intestinal tract of hospitalized patients, with only seven patients actually presenting clinical samples. Of these, four patients carried the outbreak strain and all four presented positive surveillance samples prior to the detection of clinical VREfm. The remaining three patients carried non-outbreak strains and lacked positive surveillance samples prior to the detection of clinical VREfm. Since universal screening was not implemented at our hospital, the colonization status at admission was unknown for most patients. However, we were able to identify 11 patients that flagged positive for VREfm within 48 h after admission. Of these, four patients carried non-outbreak strains (Table S1). These data suggest independent introductions of colonized patients in addition to nosocomial spread, thus it is likely that a predominant clone was already circulating locally when the outbreak was detected and, once inside, it rapidly spread from patient to patient.

In addition, just one environmental sample turned positive, suggesting poor environmental persistence and pointing to health workers and asymptomatic carriers as the most likely cause of dissemination, in good agreement with the rapid spread of the outbreak to multiple wards and units. In fact, the implementation of infection control measures, which instructed a severe reinforcement of hand hygiene and contact precautions, led to outbreak clearance in just a few months.

This is the second VREfm outbreak reported in the city of Barcelona. The previous outbreak occurred between October 2012-January 2013 at a neighboring hospital and was caused by a different lineage (ST17), affecting 13 patients [4]. However, VREfm belonging to the ST80 lineage (VanA-producing and linezolid susceptible) were recently reported in a tertiary hospital during 2018–2019 in Spain [25]. Notably, while ST80 has never been associated with the main *E. faecium* lineages circulating in Spain [26], it is recognized as one of the most common clones in European countries with much higher prevalence of VREfm [27, 28]. For instance, a linezolid-resistant ST80 VREfm carrying the linezolid-resistance gene *optrA* has been recently reported causing a hospital outbreak in Ireland [28]. Interestingly, the ST80 outbreak strain in our study as well as two additional non-outbreak ST80 isolates were also resistant to linezolid. In our study though, resistance was not due to the carriage of *optrA* but *poxtA*, which encodes an ABC-F type ribosomal protection protein that is also commonly located on conjugative plasmids, making it easily transmissible among enterococcal lineages [22]. Concomitance of resistance mechanisms within ST80 strains in our hospital implies that treatment options were rather limited, since both outbreak and non-outbreak ST80 strains were susceptible to daptomycin only.

The VREfm outbreak also provided an opportunity to assess the ability of FT-IR to rapidly detect and identify ongoing clonal dissemination and compare its performance against MLST, cgMLST, and PFGE, which are all widely accepted epidemiological methods for outbreak examination. Although FT-IR provides limited information compared to WGS techniques and while it is only useful at a local scale due to the current lack of consensus regarding the interlab exchange of FT-IR data, its rapid identification of clonal relatedness in our study proved critical for the swift implementing of infection control measures. The main advantage of FT-IR is its ability to provide almost real-time results (turnaround time of FT-IR is less than three hours) with minimal sample processing, along with ease of analysis and interpretation.

Regarding performance, cluster analysis of VREfm isolates in this study using FT-IR yielded the same results as both cgMLST and PFGE, and outperformed MLST, which showed poorer resolving power. Nevertheless, the designation of cut-off values for FT-IR clustering analysis remains subjective and relies on an experienced user and the knowledge provided by the gold-standard methods. In this study, the chosen cut-off value to compare the initial outbreak isolates (Figure 1) was set at a Euclidean distance of 0.130, without the aid of any other method. This initial cut-off value aligned perfectly with the final optimal cut-off value chosen in the performance study (Figure 4) that showed 100% agreement with both PFGE and WGS. A recent publication also identified an optimal cut-off value for the correct clustering of VREfm isolates around 0.140 [29]. Optimal cut-off values, based on our experience and on published reports, seem to be species-specific and may vary widely, with some microorganisms having cut-off values as high as 0.5. Therefore, it is crucial to gather as much data as possible for any given species to fine-tune the range of cut-off values to be used without relying on gold-standard methods. A cut-off range of 0.130-0.140 for VREfm is pretty low and most likely reflects the high degree of relatedness of hospital-acquired VREfm strains, as they all belong to the same clonal complex (CC17) and are typically single or double locus variants of each other in the MLST analysis.

The limited diversity of clones in our study could be considered a limitation, and the inclusion of a more diverse collection may impact the level of agreement between all methods. However, we believe that the purpose of FT-IR typing is not necessarily the correct clustering of every single strain within a large collection, but rather to rapidly identify clonal relatedness among locally disseminating isolates. In this regard, FT-IR technology demonstrates remarkable performance.

## Conclusions

FT-IR spectroscopy offers rapid turnaround times, sensitivity and reproducibility, delivering results comparable to both traditional and contemporary typing methods. It serves as an excellent tool for routine monitoring of clonal dissemination and early outbreak detection. Compared to WGS, it provides limited yet crucial information but at a significantly lower cost and with a faster turnaround time. The implementation of FT-IR-based surveillance at our institution led to the rapid identification of a VREfm outbreak, enabling prompt implementation of infection control measures and ultimately leading to successful containment of the outbreak.

## Supplementary Material

TableS1.pdf
