# Supplementary material for: Early identification of the nosocomial spread of vancomycin-resistant Enterococcus faecium by Fourier-transform infrared spectroscopy and performance comparison with PFGE and WGS
Source: Emerg Microbes Infect. 2024 Aug 13;13(1):2392659. doi: 10.1080/22221751.2024.2392659 (PMC11346335; doi:10.1080/22221751.2024.2392659)
Supplement: TableS1.pdf [file TEMI_A_2392659_SM1503.pdf]

Table S1. Microbiological features of 71 *E. faecium* isolates recovered from February through May 2023 at a single tertiary hospital in Barcelona.

Strain	DateI	TTD	DateA	P#	Ward	Sample	Van	Clusters					MIC (mg/L)						LZD_R
								PFGE	FTIR	ST	CC	cgMLST	AMP	VAN	TEC	LZD	IPM	DAP	
23-0119*	03/02/2023	18	16/01/2023	1	Hepa	RS	*vanA*	VRE_1	VRE_1	80	CC17	7795	>16	>8	>8	>4	>8	2	poxtA
23-0217*	06/03/2023	53	12/01/2023	2	Pneu	RS	*vanA*	VRE_1	VRE_1	80	CC17	7795	>16	>8	>8	>4	>8	2	poxtA
23-0227*	08/03/2023	0	08/03/2023	3	Hepa	RS	*vanA*	VRE_1	VRE_1	80	CC17	7795	>16	>8	>8	>4	>8	1	poxtA
23-0254*	22/03/2023	0	22/03/2023	4	Onco	RS	*vanA*	VRE_9	VRE_9	1421	CC17	6552	>16	>8	>8	1	>8	2	
23-0279	27/03/2023	11	16/03/2023	7	Trau	RS	*vanA*	VRE_1	VRE_1	80	CC17	7795	>16	>8	>8	>4	>8	2	
23-0268	28/03/2023	6	22/03/2023	4	Onco	RS	*vanA*	VRE_9	VRE_9	1421	CC17	6552	>16	>8	>8	1	>8	2	
23-0278	29/03/2023	26	03/03/2023	6	Onco	RS	*vanA*	VRE_1	VRE_1	80	CC17	7795	>16	>8	>8	>4	>8	2	
23-0277*	29/03/2023	5	24/03/2023	8	Onco	RS	*vanA*	VRE_1	VRE_1	80	CC17	7795	>16	>8	>8	>4	>8	2	poxtA
23-0276	31/03/2023	21	10/03/2023	5	Onco	RS	*vanA*	VRE_1	VRE_1	80	CC17	7795	>16	>8	>8	>4	>8	2	
23-0351	03/04/2023	23	11/03/2023	14	Onco	RS	*vanA*	VRE_1	VRE_1	80	CC17	7795	>16	>8	>8	>4	>8	2	
23-0349	03/04/2023	26	08/03/2023	15	Onco	RS	*vanA*	VRE_1	VRE_1	80	CC17	7795	>16	>8	>8	>4	>8	2	
23-0348*	04/04/2023	8	27/03/2023	10	Onco	RS	*vanA*	VRE_1	VRE_1	80	CC17	7795	>16	>8	>8	>4	>8	2	poxtA
23-0350*	04/04/2023	13	22/03/2023	4	Onco	RS	*vanA*	VRE_9	VRE_9	1421	CC17	6552	>16	>8	>8	1	>8	2	
23-0291	05/04/2023	24	12/03/2023	11	CVSurg	RS	*vanA*	VRE_1	VRE_1	80	CC17	7795	>16	>8	>8	>4	>8	2	
23-0292*	06/04/2023	21	16/03/2023	7	Pneu	RS	*vanA*	VRE_1	VRE_1	80	CC17	7795	>16	>8	>8	>4	>8	2	poxtA
23-0307	08/04/2023	29	10/03/2023	5	Onco	Wd	*vanA*	VRE_1	VRE_1	80	CC17	7795	>16	>8	>8	>4	>8	2	

Strain	DateI	TTD	DateA	P#	Ward	Sample	Van	Clusters					MIC (mg/L)						LZD_R
								PFGE	FTIR	ST	CC	cgMLST	AMP	VAN	TEC	LZD	IPM	DAP	
23-0289*	09/04/2023	2	07/04/2023	9	Onco	RS	*vanA*	VRE_1	VRE_1	80	CC17	7795	>16	>8	>8	>4	>8	1	*poxtA*
23-0312*	11/04/2023	9	02/04/2023	12	Hepa	RS	*vanA*	VRE_1	VRE_1	80	CC17	7795	>16	>8	>8	>4	>8	1	*poxtA*
23-0317	11/04/2023	76	25/01/2023	13	Onco	RS	*vanA*	VRE_1	VRE_1	80	CC17	7795	>16	>8	>8	>4	>8	2	
23-0318	11/04/2023	31	11/03/2023	14	Onco	RS	*vanA*	VRE_1	VRE_1	80	CC17	7795	>16	>8	>8	>4	>8	2	
23-0454	11/04/2023	8	03/04/2023	40	Onco	RS	*vanA*	VRE_1	VRE_1	80	CC17	7795	>16	>8	>8	>4	>8	2	
23-0319	11/04/2023	32	10/03/2023	5	Onco	RS	*vanA*	VRE_1	VRE_1	80	CC17	7795	>16	>8	>8	>4	>8	2	
23-0329	13/04/2023	15	29/03/2023	16	Onco	RS	*vanA*	VRE_1	VRE_1	80	CC17	7795	>16	>8	>8	>4	>8	2	
23-0330	13/04/2023	2	11/04/2023	17	Onco	RS	*vanA*	VRE_1	VRE_1	80	CC17	7795	>16	>8	>8	>4	>8	2	
23-0346	18/04/2023	4	14/04/2023	54	Onco	RS	*vanA*	VRE_1	VRE_1	80	CC17	7795	>16	>8	>8	>4	>8	2	
23-0336	19/04/2023	27	23/03/2023	18	Onco	RS	*vanA*	VRE_1	VRE_1	80	CC17	7795	>16	>8	>8	>4	>8	2	
23-0337	19/04/2023	19	31/03/2023	19	Onco	RS	*vanA*	VRE_1	VRE_1	80	CC17	7795	>16	>8	>8	>4	>8	2	
23-0338	19/04/2023	17	02/04/2023	20	Onco	RS	*vanA*	VRE_1	VRE_1	80	CC17	7795	>16	>8	>8	>4	>8	2	
23-0455	20/04/2023	13	07/04/2023	9	Onco	RS	*vanA*	VRE_1	VRE_1	80	CC17	7795	>16	>8	>8	>4	>8	2	
23-0374*	24/04/2023	47	08/03/2023	15	Onco	Wd	*vanA*	VRE_1	VRE_1	80	CC17	7795	>16	>8	>8	>4	>8	1	
23-0363*	25/04/2023	48	08/03/2023	15	Onco	RS	*vanA*	VRE_1	VRE_1	80	CC17	7795	>16	>8	>8	>4	>8	1	*poxtA*
23-0371*	25/04/2023	3	22/04/2023	22	InfDis	RS	*vanA*	VRE_1	VRE_1	80	CC17	7795	>16	>8	>8	>4	>8	2	*poxtA*
23-0369	25/04/2023	46	10/03/2023	5	Onco	RS	*vanA*	VRE_1	VRE_1	80	CC17	7795	>16	>8	>8	>4	>8	2	
23-0368*	26/04/2023	2	24/04/2023	21	Onco	Bd	Neg	VRE_4	VRE_4	117	CC17	6173	>16	0,5	0,5	1	>8	2	
23-0385*	27/04/2023	1	26/04/2023	24	IntMed	RS	*vanA*	VRE_1	VRE_1	80	CC17	7795	>16	>8	>8	>4	>8	2	*poxtA*

Strain	DateI	TTD	DateA	P#	Ward	Sample	Van	Clusters					MIC (mg/L)						LZD_R
								PFGE	FTIR	ST	CC	cgMLST	AMP	VAN	TEC	LZD	IPM	DAP	
23-0403	27/04/2023	6	21/04/2023	28	InfDis	RS	*vanA*	VRE_1	VRE_1	80	CC17	7795	>16	>8	>8	>4	>8	2	
23-0384	28/04/2023	13	15/04/2023	23	InfDis	RS	*vanA*	VRE_1	VRE_1	80	CC17	7795	>16	>8	>8	>4	>8	2	
23-0386	28/04/2023	2	26/04/2023	25	Onco	RS	*vanA*	VRE_1	VRE_1	80	CC17	7795	>16	>8	>8	>4	>8	2	
23-0387	28/04/2023	16	12/04/2023	26	InfDis	RS	*vanA*	VRE_1	VRE_1	80	CC17	7795	>16	>8	>8	>4	>8	2	
23-0392	28/04/2023	9	19/04/2023	27	InfDis	RS	*vanA*	VRE_1	VRE_1	80	CC17	7795	>16	>8	>8	>4	>8	2	
23-0381*	28/04/2023	NA	NA	45	DomH	Wd	Neg	VRE_3	VRE_3	80	CC17	7798	>16	0,5	0,5	>4	>8	2	*poxtA*
23-0407	03/05/2023	13	20/04/2023	29	DomH	RS	*vanA*	VRE_1	VRE_1	80	CC17	7795	>16	>8	>8	>4	>8	2	
23-0426*	04/05/2023	87	06/02/2023	30	DomH	Wd	*vanB*	VRE_8	VRE_8	2446	CC17	7796	>16	>8	1	2	>8	1	
23-0427	04/05/2023	23	11/04/2023	31	Onco	RS	*vanA*	VRE_1	VRE_1	80	CC17	7795	>16	>8	>8	>4	>8	2	
23-0428*	04/05/2023	14	20/04/2023	32	SICU	RS	*vanA*	VRE_1	VRE_1	80	CC17	7795	>16	>8	>8	>4	>8	2	*poxtA*
23-0432	04/05/2023	68	25/02/2023	34	Onco	RS	*vanA*	VRE_1	VRE_1	80	CC17	7795	>16	>8	>8	>4	>8	2	
23-0433	04/05/2023	40	25/03/2023	35	Onco	RS	*vanA*	VRE_1	VRE_1	80	CC17	7795	>16	>8	>8	>4	>8	2	
23-0434*	04/05/2023	15	19/04/2023	36	Onco	RS	*vanB*	VRE_5	VRE_5	117	CC17	7799	>16	2	1	1	>8	2	
23-0431	05/05/2023	40	26/03/2023	33	InfDis	RS	*vanA*	VRE_1	VRE_1	80	CC17	7795	>16	>8	>8	>4	>8	2	
23-0435	08/05/2023	31	07/04/2023	37	Pneu	RS	*vanA*	VRE_1	VRE_1	80	CC17	7795	>16	>8	>8	>4	>8	2	
23-0445	08/05/2023	NA	NA	38	InfDis	Env	*vanA*	VRE_1	VRE_1	80	CC17	7795	>16	>8	>8	>4	>8	2	
23-0450	10/05/2023	5	05/05/2023	39	SICU	RS	*vanA*	VRE_1	VRE_1	80	CC17	7795	>16	>8	>8	>4	>8	2	
23-0463	11/05/2023	4	07/05/2023	41	Onco	RS	*vanA*	VRE_1	VRE_1	80	CC17	7795	>16	>8	>8	>4	>8	2	
23-0464	11/05/2023	27	14/04/2023	42	Onco	RS	*vanA*	VRE_1	VRE_1	80	CC17	7795	>16	>8	>8	>4	>8	2	

Strain	DateI	TTD	DateA	P#	Ward	Sample	Van	Clusters					MIC (mg/L)						LZD_R
								PFGE	FTIR	ST	CC	cgMLST	AMP	VAN	TEC	LZD	IPM	DAP	
23-0476	11/05/2023	5	06/05/2023	44	IntMed	RS	*vanA*	VRE_1	VRE_1	80	CC17	7795	>16	>8	>8	>4	>8	2	
23-0474	12/05/2023	21	21/04/2023	28	InfDis	RS	*vanA*	VRE_1	VRE_1	80	CC17	7795	>16	>8	>8	>4	>8	2	
23-0488	15/05/2023	1	14/05/2023	46	SICU	RS	*vanA*	VRE_1	VRE_1	80	CC17	7795	>16	>8	>8	>4	>8	2	
23-0500*	19/05/2023	0	19/05/2023	47	SICU	RS	*vanA*	VRE_6	VRE_6	18	CC17	7800	>16	>8	>8	2	>8	2	
23-0489*	20/05/2023	103	06/02/2023	30	DomH	RS	*vanB*	VRE_8	VRE_8	2446	CC17	7796	>16	>8	1	2	>8	1	
23-0505*	21/05/2023	0	21/05/2023	48	IntMed	RS	*vanA*	VRE_10	VRE_10	80	CC17	5967	>16	>8	>8	4	>8	2	*poxtA*
23-0533*	24/05/2023	5	19/05/2023	47	SICU	RS	*vanA*	VRE_2	VRE_2	17	CC17	7801	>16	>8	>8	1	>8	1	
23-0534	24/05/2023	0	24/05/2023	51	ER	RS	*vanA*	VRE_1	VRE_1	80	CC17	7795	>16	>8	>8	>4	>8	2	
23-0531*	25/05/2023	9	16/05/2023	49	CVSurg	RS	*vanA*	VRE_7	VRE_7	80	CC17	847	>16	>8	>8	>4	>8	4	*poxtA*
23-0532*	25/05/2023	5	20/05/2023	50	CVSurg	RS	*vanA*	VRE_1	VRE_1	80	CC17	7795	>16	>8	>8	>4	>8	2	*poxtA*
23-0537	26/05/2023	62	25/03/2023	35	Onco	RS	*vanA*	VRE_1	VRE_1	80	CC17	7795	>16	>8	>8	>4	>8	2	
23-0544	30/05/2023	34	26/04/2023	24	IntMed	RS	*vanA*	VRE_1	VRE_1	80	CC17	7795	>16	>8	>8	>4	>8	2	
23-0552	30/05/2023	57	03/04/2023	40	ThorSurg	RS	*vanA*	VRE_1	VRE_1	80	CC17	7795	>16	>8	>8	>4	>8	2	
23-0543	30/05/2023	6	24/05/2023	52	Hepa	RS	*vanA*	VRE_1	VRE_1	80	CC17	7795	>16	>8	>8	>4	>8	2	
23-0553*	31/05/2023	17	14/05/2023	46	Uro	Wd	*vanA*	VRE_1	VRE_1	80	CC17	7795	>16	>8	>8	>4	>8	1	
23-0551	31/05/2023	15	16/05/2023	53	IntMed	RS	*vanA*	VRE_1	VRE_1	80	CC17	7795	>16	>8	>8	>4	>8	2	
23-0601*	31/05/2023	35	26/04/2023	55	Onco	RS	*vanB*	VRE_5	VRE_5	117	CC17	7799	>16	2	1	2	>8	4	

Strain: Strain designation; DateI: Date of isolation; TTA: Time to detection; DateA: Date of admission; P#: Patient number; Ward: Ward of isolation; Sample: Sample type; van: Positive for *vanA/vanB* genes; PFGE: Pulsed-field gel electrophoresis; FTIR: Fourier-transform Infrared spectroscopy;

ST: Sequence type; CC: Clonal Complex; cgMLST: Core genome multi-locus sequence typing; MIC: Minimum inhibitory concentration; AMP: Ampicillin; VAN: vancolycin; TEC: Teicoplanin; LZD: Linezolid; IPM: Imipenem; DAP: Daptomycin; LZD_R; Linezolid resistance. Hepa: Hepatology; Pneu: Pneumology; Onco: Onco-hematology; Trau: Traumatology; CVSurg: Cardiovascular surgery; IntMed: Internal medicine; InfDis: Infectious diseases; DomH: Domiciliary hospitalization; SICU: Surgery ICU; ER: Emergency room; ThorSurg: Thoracic surgery; Uro: Urology; RS: Rectal Swab; Wd: Wound; Bd: Blood sample: Env: Environmental sample; NA: Not applicable. Cells in red show resistance while those in green indicate susceptibility, according to the EUCAST guidelines and breakpoints for *Enterococcus* (European Committee on Antimicrobial Susceptibility Testing, 2023 v.13). *Isolates selected for whole genome sequencing.
